# Rescue intrauterine insemination in women failing to retrieve oocytes at ovum pick-up: report of a case series

**DOI:** 10.1007/s10815-024-03091-z

**Published:** 2024-04-18

**Authors:** Ludovico Muzii, Giulia Galati, Ilenia La Barbiera, Antonella Linari, Oriana Capri, Daniela Pietrangeli, Edgardo Somigliana

**Affiliations:** 1 Department of Maternal and Child Health and Urological Sciences, Sapienza University of Rome, 00161 Rome, Italy; 2 Department of Clinical Sciences and Community Health, University of Milan, Milan, Italy; 3 Infertility Unit, Fondazione IRCCS Ca’ Granda Ospedale Maggiore Policlinico, Milan, Italy

**Keywords:** Intrauterine insemination, IVF, Oocyte, Pick-up

## Abstract

**Purpose:**

Failure to collect oocytes at the time of oocyte pick-up is an unfavorable outcome of in vitro fertilization (IVF) cycles. In these cases, prompt intrauterine insemination (IUI) could be an option (rescue IUI), but this possibility has been poorly studied.

**Methods:**

Rescue IUI is routinely offered in our unit in women failing to retrieve oocytes, provided that they have at least one patent tube, normal male semen analysis, and the total number of developed follicles is ≤ 3. We therefore reviewed all oocyte retrievals performed from 2006 to 2022 in our unit to identify these cases. As a comparator, we referred to preplanned IUI performed during the same study period. The 95% confidence interval (95% CI) of proportions was calculated using a binomial distribution model.

**Results:**

Rescue IUI was performed in 96 out of 3531 oocyte retrievals (2.7%; 95% CI 2.2–3.3%). Six live births were obtained, corresponding to 6.2% (95% CI 2.3–13.1). All pregnancies were singletons.

**Conclusions:**

Rescue IUI in women failing to retrieve oocytes is a possible option that may be considered in selected cases. The efficacy is low, but the procedure is simple, and without significant risks. Generalizability to a conventional IVF protocol setting is however limited.

## Introduction

Failure to collect oocytes at the time of oocyte pick-up, despite a presumed correct timing of the human chorionic gonadotropin (hCG) trigger, is an unfavorable outcome of in vitro fertilization (IVF) cycles [1]. This rare event is more common in poor responders and in mild stimulation cycles, due to the lower number of follicles available for oocyte pick-up (OPU) in these circumstances [1].

When this event occurs, the most common option is to cancel any further step, since the absence of available oocytes obviously precludes IVF or intracytoplasmic sperm injection (ICSI) and subsequent embryo transfer (ET). An alternative reasonable option is to proceed to intrauterine insemination (IUI), if at least one tube is patent, and if the male partner has no major alteration of the seminal variables, in the hypothesis that the oocytes released in the peritoneal fluid or retained in the follicle may be picked up by the fimbriated ends of the tube after the pick-up procedure. Very limited data are present in the literature on this “rescue IUI” procedure after a failed OPU. A single study by Matorras et al. explored this option in low responder patients, but failed to observe any pregnancy among 54 treated cases (0%, 95% CI 0.0–6.8%) [2].

Rescue IUI in case of failed OPU is systematically performed in our unit when no oocytes are retrieved. Given the paucity of available evidence in the literature and the simplicity of the procedure, we deemed of relevance reporting on the results of this approach in our setting.

## Materials and methods

Oocyte retrievals performed from January 1, 2006, to December 31, 2022, at the Infertility Unit of Policlinico Umberto I, Sapienza University of Rome, Italy, were reviewed. We identified women who failed to retrieve any oocyte and who performed rescue IUI. In addition, all pre-planned IUI performed during the same study period were reviewed. The aim was having a comparator. The study was accepted by the local Institutional Review Board (IRB). An informed consent was not requested because this is a retrospective study. However, all patients referring to our unit sign an informed consent for their data to be used for research purposes.

During the study period, ovarian stimulation was performed with human menopausal gonadotropins (hMG) (Meropur, Ferring), within a flexible GnRH antagonist protocol (Fyremadel, Ferring). Given the restrictive Italian legislation (the number of obtained embryos per cycle should not exceed three), the policy of ovarian stimulation adopted in our setting aimed at a low number of oocytes, i.e., between 2 and 7 [3]. The starting hMG dose varied therefore from 75 to 225 UI, depending on the age of the patient, and on anti-Mullerian hormone (AMH) levels. When at least two follicles reach a diameter of 17 mm, triggering for final oocyte maturation was administered with 10.000 IU of human chorionic gonadotropin (hCG) (Gonasi HP, IBSA).

Oocyte retrieval was performed 36 h after hCG administration. ET was performed either on day 3 or day 5 of embryo development. Luteal supplementation was provided with micronized vaginal progesterone, 200 mg three times daily, starting from the day of ET.

During the study period, women who failed to retrieve any oocyte at oocyte retrieval could be converted to IUI, when permitting conditions were present. Permitting conditions were considered as follows: at least one patent tube at either hysterosonosalpingography or hysterosalpingography, normal male semen analysis according to WHO criteria [4–6], normal motile sperm count after sperm preparation (> 3 million/mL), total number of developed follicles ≤ 3, and informed consent of the couple, particularly addressing the possibility, although remote, of a twin or higher order pregnancy in case of two or three follicles > 17 mm. The IUI procedure was performed approximately 1 h after the failed oocytes retrieval, as described in detail elsewhere [7].

Luteal supplementation was provided with micronized vaginal progesterone, 200 mg three times daily, as for IVF, initiating 3 days later.

Data of the included women were obtained from patients’ charts. The 95% confidence interval (95% CI) of proportions was calculated using a binomial distribution model. Characteristics of women performing rescue IUI and pre-planned IUI were compared using Student’s *t* test, Mann–Whitney test, chi-square test, or Fisher exact test, as appropriate. A pre-planned sample size calculation was not performed, but we estimated that the available cases could allow us to provide an estimate of the rate of success of the procedure with a 95% CI of about ± 5%.

## Results

Overall, 3531 oocyte retrievals were performed during the study period. A total of 96 fulfilled the criteria for rescue IUI, and all eligible women accepted to undergo the procedure. Overall, the rate of rescue IUI was 2.7% (95% CI 2.2–3.3%). The mean number of follicles > 17 mm at the time of trigger was 1.85, proving an ovarian response lower than expected in a mild stimulation protocol, which aimed at two to seven follicles. Thirty-seven patients out of the 96 patients (38.5%) had less than two follicles larger than 17 mm at the time of trigger. None of the 96 patients could be defined as poor responders before ovulation induction, based on the definition of AMH < 0.5 ng/mL [8].

The IUI procedure was successful in all cases, with no complications. All patients followed the prescribed luteal phase support protocol. A total of six clinical pregnancies occurred, corresponding to 6.2% (95% CI 2.3–13.1). All pregnancies obtained after the rescue IUI procedure progressed uneventfully, and all patients delivered healthy newborns at term. The rate (95% CI) of live birth is therefore identical to the clinical pregnancy rate. All pregnancies were singletons.

In Table 1, the patient characteristics of the rescue IUI group were compared the control group of pre-planned IUIs that were performed during the same study period. As expected, some statistically significant differences emerged. However, the chance of success did not differ. In women undergoing scheduled IUIs, 67 clinical pregnancies (6.1%) and 62 live birth rate (5.6%) were obtained out of 1108 procedures performed. These rates were not different from what observed in rescue IUI (*p* = 0.93 and *p* = 0.97, for clinical pregnancy and live birth rates, respectively). We emphasize the fact that the pregnancy rate for both groups (6.2% and 6.1%) are lower than commonly reported [9, 10].

**Table 1:** Demographic and cycle characteristics of rescue intrauterine insemination and general intrauterine insemination populations (data are express as mean ± SD)

	Rescue IUI	Pre-planned IUI	*p* value
Number of cycles	96	1108	
Woman’s age (years; mean SD)	37.64 ± 3.79	36.01 ± 4.53	0.005
Days of stimulation	9.7 ± 3.87	7.55 ± 3.54	0.02
Number of follicles > 17 mm	1.85 ± 1.18	1.01 ± 0.74	0.003
Estradiol levels on the day of HCG administration	883.66 ± 834.14	595.36 ± 636.91	0.030

## Discussion

Rescue IUI in women failing to retrieve oocytes and whose clinical conditions do not contraindicate IUI is a possible option. In our setting, the clinical conditions allowing this procedure was rare (2.7%, corresponding to one in 37 oocytes retrievals) and the success rate was low (6.2%, 95% CI 2.3–13.1%). However, it must be underlined that the success rate was like what observed in pre-planned IUI cycles, that all procedures were uncomplicated, and that all obtained pregnancies were singletons. Of relevance here is that these pregnancies must be considered additional successes since, in the absence of the rescue-IUI, the women would have had no chance to becoming pregnant, except for the possibility of achieving a pregnancy with intercourse after the procedure. On the other hand, we must emphasize the low pregnancy rates in both groups.

Failure to obtain oocytes at the time of egg retrieval is uncommon. This event is reported in 0.05 to 7% of conventional IVF/ICSI cycles [11]. In 5–20% of dominant follicles, no oocytes can be obtained [12]. This event most commonly occurs if oocyte retrieval is not correctly timed with respect to hCG administration [13]. After hCG administration, only serum assessments of hCG and progesterone levels can prove the correct trigger timing.

However, this event may also occur after correct timing of hCG trigger, possibly because a cumulo-corona complex does not detach from the follicle wall during OPU, or if it is released in the peritoneal fluid instead of being aspirated.

There are two different scenarios in which the event of no oocyte retrieved may occur more frequently despite a presumably correct timing of hCG triggering: in poor responders after a conventional ovarian stimulation cycle or in mild stimulation cycles. In both scenarios, in fact, the lower number of large follicles available exposes the patient to a higher risk of not collecting oocytes simply as a product of chance: the lower the number of punctured follicles, the higher the possibility of not retrieving any oocyte at the time of follicle aspiration. However, poor responders and mild stimulation cases may differ. Alongside with the lower chance of retrieving oocytes due to the product of chance in case of low number of follicles, i.e., a quantity issue, in case of low responders, one may hypothesize an additional risk, which is an impaired folliculogenesis, which puts the patient in an unexpected low rate of collected oocytes, i.e., a quality issue.

Generally, in cases where no oocytes are collected at OPU, the ART procedure is aborted, and no further action is undertaken. This option is obviously frustrating for both the patients and the physician. However, it is also possible that the oocyte, if present in the follicle, may be later picked-up by the fimbriated end of the tube. Therefore, attempts at spontaneous conception may be a reasonable option, if at least one tube is patent, if there is no major male factor of infertility, and if the number of follicles at the time of hCG trigger is not too high, to avoid the risk of high-order pregnancies. A third option, besides simply abort the procedure, or shift to spontaneous conception with timed intercourse, is to resort to a “rescue” IUI, which has the advantage of being practical and patient friendly, since the patient is already in the facility, and the seminal fluid has just been prepared for the planned IVF/ICSI. However, even in the scenario of “rescue IUI” reported in the present study, a possible delayed natural ovulation and conception cannot be excluded.

Our results, the present case series of rescue IUI, contrast with the unique available evidence in the literature on rescue-IUI [2]. These authors failed to document any success out of 54 poor responder patients (0%, 95% CI 0–6.7%). The procedure was performed when no oocytes were retrieved at the time of OPU, despite three or more follicles over 18 mm were present at the time of trigger. The 0% pregnancy rate obtained after rescue IUI was significantly inferior to the 17.5% rate obtained after 5394 regularly scheduled IUI. Some differences in the studied population may explain the inconsistency with our findings. Of utmost relevance here is the systematic policy of mild stimulation applied in our unit, guided by the objective of obtaining less than seven oocytes. In contrast to Matorras et al., we did not include poor responders in our study, but predicted normal responders treated with a low dose of gonadotropins. This latter population could be at better prognosis also for IUI success. A second possible explanation for the inconsistency between the two studies is related to the statistical power. None of the study was sufficiently powered to provide a precise estimate of the rate of success. Noteworthy, the 95% CIs of the pregnancy rates of the two studies largely overlap (2.3–13.1% in our hands and 0–6.7% for Matorras et al.), and we cannot therefore exclude that differences may be only secondary to statical fluctuations.

Noteworthy, if one combines the two studies (6 successes out of 96 + 54 treated subjects), the rate of success would be 4.0% (95% CI 1.5–8.5%).

Some limitations must be acknowledged for the present study: the limited simple size for the rescue IUI group, the low pregnancy rates in both groups, and the inclusion of patients submitted to a mild stimulation protocol, which is not the routine approach in IVF clinics, where the aim is normally to achieve up to 15 oocytes [14]. Consequently, the generalizability to a conventional IVF protocol setting is however limited, due to the risk of high order multiple pregnancies. Also, a false empty follicle syndrome with delayed ovulation cannot be ruled out in your population as serum confirmation of hCG administration was not part of our clinical practice.

In conclusion, rescue IUI in women failing to retrieve oocytes is a possible option that may be considered in selected cases. The efficacy is low, but the procedure is simple, and the absence of significant risks should receive utmost consideration when balancing the pros and cons with the patients. However, further studies are needed to confirm these data.
